# Volumetric assessment of the periablational safety margin after thermal ablation of colorectal liver metastases

**DOI:** 10.1007/s00330-020-07579-x

**Published:** 2021-01-14

**Authors:** Gregor Laimer, Nikolai Jaschke, Peter Schullian, Daniel Putzer, Gernot Eberle, Marco Solbiati, Luigi Solbiati, S. Nahum Goldberg, Reto Bale

**Affiliations:** 1 Interventional Oncology-Microinvasive Therapy (SIP), Department of Radiology, Medical University Innsbruck, Anichstr. 35, 6020 Innsbruck, Austria; 2 Department of Internal Medicine I, Gastroenterology, Hepatology, Endocrinology and Metabolism, Medical University Innsbruck, Anichstr. 35, 6020 Innsbruck, Austria; 3 Division of Endocrinology and Metabolic Bone Diseases, Department of Medicine III, Technical University of Dresden, Fetscherstr. 74, 01037 Dresden, Germany; 4 R&D Unit, R.A.W. Srl, Milan, Italy; 5 Department of Biomedical Sciences, Humanitas University, Via Rita Levi Montalcini 4, 20072 Pieve Emanuele, Milan, Italy; 6 IRCCS Humanitas Research Hospital, via Manzoni 56, 20089 Rozzano, Milan, Italy; 7 Department of Radiology, Hadassah Hebrew University Medical Centre, Jerusalem, Israel; 8 Department of Radiology, Beth Israel Deaconess Medical Center, Boston, MA USA

**Keywords:** Radiofrequency ablation, Liver neoplasms, Tomography, X-ray computed, Treatment outcome

## Abstract

**Objectives:**

To retrospectively assess the periablational 3D safety margin in patients with colorectal liver metastases (CRLM) referred for stereotactic radiofrequency ablation (RFA) and to evaluate its influence on local treatment success.

**Methods:**

Forty-five patients (31 males; mean age 64.5 [range 31–87 years]) with 76 CRLM were treated with stereotactic RFA and retrospectively analyzed. Image fusion of pre- and post-interventional contrast-enhanced CT scans using a non-rigid registration software enabled a retrospective assessment of the percentage of predetermined periablational 3D safety margin and CRLM successfully ablated. Periablational safety zones (1–10 mm) and percentage of periablational zone ablated were calculated, analyzed, and compared with subsequent tumor growth to determine an optimal safety margin predictive of local treatment success.

**Results:**

Mean overall follow-up was 36.1 ± 18.5 months. Nine of 76 CRLMs (11.8%) developed local tumor progression (LTP) with mean time to LTP of 18.3 ± 11.9 months. Overall 1-, 2-, and 3-year cumulative LTP-free survival rates were 98.7%, 90.6%, and 88.6%, respectively. The periablational safety margin assessment proved to be the only independent predictor (*p* < 0.001) of LTP for all calculated safety margins. The smallest safety margin 100% ablated displaying no LTP was 3 mm, and at least 90% of a 6-mm circumscribed 3D safety margin was required to achieve complete ablation.

**Conclusions:**

Volumetric assessment of the periablational safety margin can be used as an intraprocedural tool to evaluate local treatment success in patients with CRLM referred to stereotactic RFA. Ablations achieving 100% 3D safety margin of 3 mm and at least 90% 3D safety margin of 6 mm can predict treatment success.

**Key Points:**

*• Volumetric assessment of the periablational safety margin can be used as an intraprocedural tool to evaluate local treatment success following thermal ablation of colorectal liver metastases.*

*• Ablations with 100% 3D periablational safety margin of 3 mm and ablations with at least 90% 3D safety margin of 6 mm can be considered indications of treatment success.*

*• Image fusion of pre- and post-interventional CT scans with the software used in this study is feasible and could represent a useful tool in daily clinical practice.*

## Introduction

Colorectal cancer is the second leading cause of cancer-related death in developed countries [1]. Approximately 25–30% of affected individuals will eventually develop colorectal liver metastases (CRLM) during their disease course [2, 3]. Radiofrequency ablation (RFA) has emerged as an alternative, potentially curative approach in the treatment of CRLM. RFA is usually tissue sparing and rarely evokes major complications, and treatment-associated mortality is exceedingly low [4]. Despite numerous advantages, RFA is still not considered the treatment of choice for CRLM by most clinicians, which in part can be attributed to initial reports of local tumor progression (LTP) rates as high as 48% [5–7]. Moreover, until recently, treatment efficacy of RFA could only be defined through either the absence of a residual tumor at first follow-up (primary technical efficacy) or the absence of local tumor progression (LTP) at subsequent follow-up. As such, an immediate, intraprocedural method to predict the local treatment success following ablation of CRLM would be highly desirable, due to its potential clinical impact. Such an approach would indicate the need for and provide the possibility to perform extension of the ablation zone during the same session, if needed.

Several studies have reported that the periablational safety margin, defined as the shortest distance between tumor border and margin of the necrosis zone [4], independently predicts the LTP in CRLM, whereby ablations with safety margins > 5–10 mm exhibit lower LTP rates [7–9]. In conventional CT- or US-guided RFA, the creation of large necrosis zones extending 5–10 mm beyond tumor borders is often hampered by technical limitations, especially for larger tumors (i.e., > 3 cm), which may result in LTP ranging from 4 to 70% [10]. In contrast, these limitations have been overcome in some centers with stereotactic RFA (SRFA), a multiple-needle approach using 3D treatment planning, stereotactic needle placement and image fusion for intraoperative assessment of the periablational safety margin [11, 12].

In many institutions, the evaluation of the periablational safety margin is performed manually through side-by-side juxtaposition of pre- and post-interventional CT scans. However, this method is challenging, even for experienced radiologists, and may introduce several potential sources of error. Image fusion of pre- and post-interventional CT scans with volumetric assessment of the periablational safety margin has shown promise in overcoming such difficulties [13–15]. Nevertheless, it remains unclear whether lower percentages (e.g., 90%) of the volume of a given safety margin may also be sufficient to avoid LTP. This is especially relevant in cases where a 100% safety margin cannot be achieved for many reasons such as proximity of large vessels to the tumor [10].

The primary aim of this study was to retrospectively assess the periablational safety margin in a cohort of CRLM patients treated with stereotactic RFA through image fusion of pre- and post-interventional CT scans using a novel non-rigid registration software and evaluate its influence on LTP. An additional goal was to evaluate calculated volumes of coverage of the tumor and periablational margin and determine their impact on LTP in order to devise an objective tool to enable immediate real-time determination of the extent and percentage of safety margin requiring ablation that can reliably predict ablation outcome success.

## Material and methods

### Patients

**Fig. 1:**
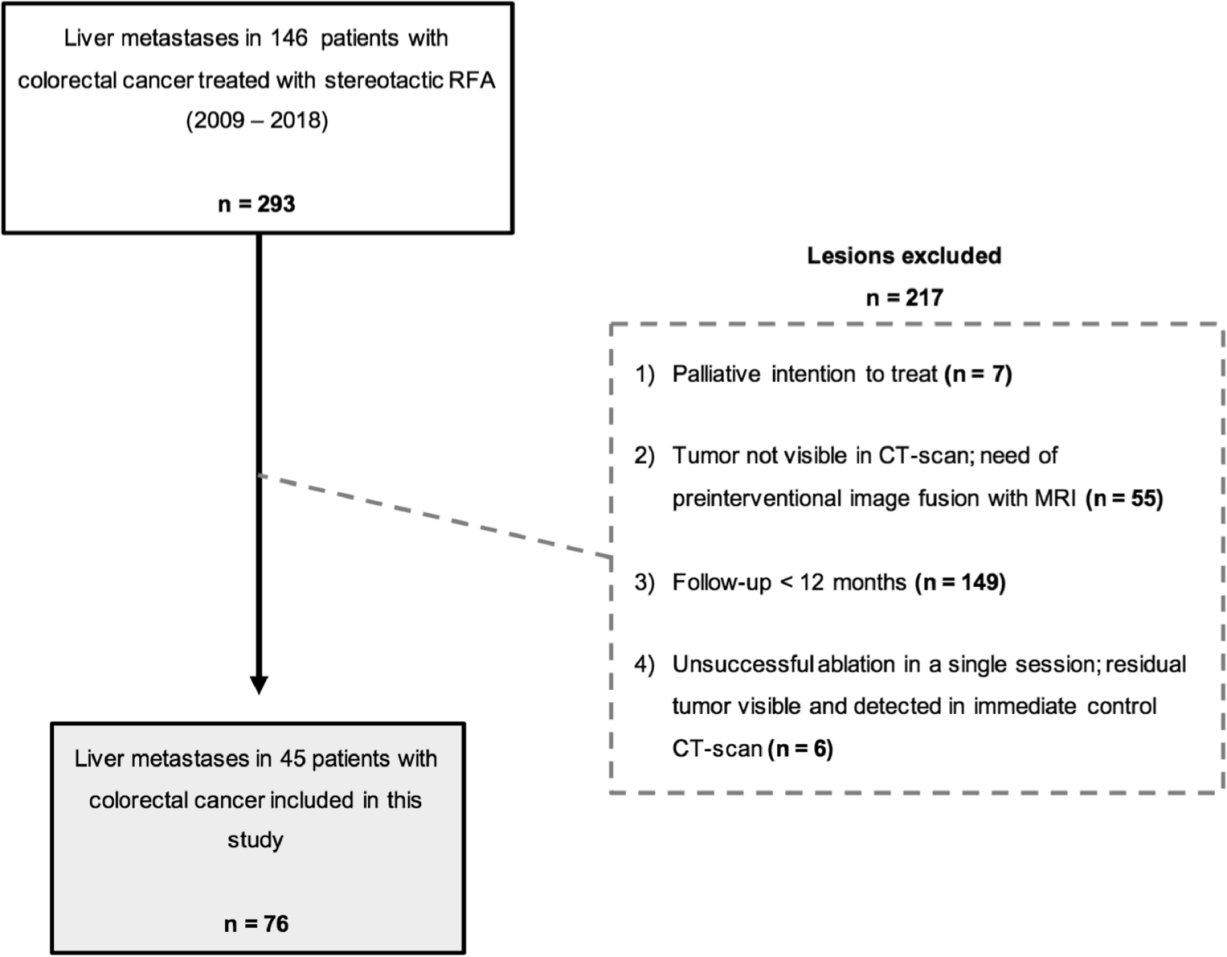
Flowchart of exclusion criteria for the evaluation of treatment success leading to 76 CRLM in 45 patients

This study was approved by the Institutional Review Board of the Medical University Innsbruck and written informed consent was obtained from all patients. Retrospective analysis of prospectively collected data from patients with up to four CRLMs identified 146 patients with 293 tumors referred to stereotactic RFA at the Department of Radiology between January 2009 and January 2018. Seventy-six liver metastases in 45 patients with colorectal cancer were deemed eligible for enrollment in this retrospective evaluation of treatment success after applying the following exclusion criteria: (1) palliative intention to treat; (2) tumor not visible in CT scan—need of pre-interventional image fusion with MRI; (3) follow-up less than 12 months; and (4) unsuccessful ablation in a single session—residual tumor visible and detected in immediate control CT scan (Fig. 1).

In all patients, the treatment plan was established at a multidisciplinary tumor board consisting of hepatologists, oncologists, liver surgeons, radiation therapists, and interventional radiologists. Treatment choice was based on tumor characteristics, liver function, anatomical considerations, and the general condition of the respective patient.

Patients could not receive stereotactic RFA if they exhibited a platelet count of less than 50,000 cells/mm^3^ or a prothrombin time ratio < 50% (prothrombin time with international normalized ratio, G1.7). Due to the multiprobe approach in stereotactic RFA, tumor size did not represent a limiting and/or exclusion factor [11, 16]. CRLM diagnosis was confirmed by typical imaging appearances on multiphasic contrast MRI or CT, with histopathological confirmation before or during stereotactic RFA procedure.

### Stereotactic radiofrequency ablation

All CRLM included in this study were treated with stereotactic RFA at a single institution at the Department of Radiology of the Medical University Innsbruck. Full procedural details of stereotactic RFA have already been described in detail [17–19] elsewhere. In brief, stereotactic RFA is performed in an interventional CT suite under general anesthesia with deep muscle relaxation. Respiratory triggering is achieved by temporary disconnection of the endotracheal tube. A dual-phase contrast-enhanced planning CT (SOMATOM Sensation Open, Siemens Inc.) with 3-mm slice thickness is obtained and the data is transferred to the optical-based 3D navigation system (Stealth Station Treon plus, Medtronic Inc.). Multiple trajectories are determined on multiplanar reformatted images in order to cover the entire tumor volume with an appropriate safety margin. Following registration, accuracy check and sterile draping, the Atlas aiming device (Interventional Systems Inc.) is adjusted by using the 3D navigation system and 15G/17.2 cm coaxial needles (Bard Inc.) are advanced through the aiming device to the target. For verification of correct needle placement, a non-enhanced CT scan is obtained and fused with the planning CT. After taking of biopsies through the coaxial needles, up to three Cool-tip RF electrodes (Medtronic Inc.) are introduced through the coaxial needles for serial tumor ablation. Needle track ablation is performed during every probe repositioning and final probe removal. Finally, a dual-phase contrast-enhanced CT scan is superimposed to the planning CT using the navigation system’s rigid registrations software in order to assess complications and the ablation result.

### Follow-up

Ablations were deemed technically successful (i.e., no evidence of residual tumor tissue) if the ablation zone covered the tumor including a 5-mm periablational safety margin, as manually measured on fused intraprocedural contrast-enhanced pre- and post-ablation datasets using the rigid registration algorithm of the navigation system. All tumors that were determined technically unsuccessful at the time of the intervention were excluded from this analysis (Fig. 1). For ablations deemed technically successful, follow-up consisted of contrast-enhanced CT or MR scans at 1-month and at 3-month intervals thereafter.

CT scans were obtained with 3-mm slice thickness; 60–70 s after initiation of contrast material injection, representing the portal venous phase. The standard protocol for contrast-enhanced MR-scans was axial T1w VIBE-DIXON (in-phase and out-of-phase); post-contrast axial T1w VIBE-DIXON in arterial (10–20 to 25–35 s), portal venous (30–45 to 90 s), delayed phase (> 2 to 4–5 min), and hepatobiliary delayed phase (> 5 to 10 min) after injection of 0.1 ml/kg contrast media (Gadobutrol 1.0 mmol/ml [Gadovist; Bayer]); axial/coronal T2w HASTE; axial T2w fat-saturated; and axial DWI (b50/400/1000). Newly detected tumors within or immediately adjacent to the necrosis zone at follow-up were defined as local tumor progression (LTP). In patients with LTP, follow-up ended at the date of its detection (i.e., occurrence of the event). Newly detected tumors distant to the ablation zone were defined as distant tumor recurrence. Only the treated tumors were evaluated as new tumor formation distant to the ablation zone did not impact upon local tumor progression, the primary endpoint of this study. Images were evaluated by two board-certified abdominal radiologists with more than 10 years of experience by consensus (B.R. and P.E.S.).

### Image fusion and evaluation of the periablational safety margin

Image fusion of pre- and post-interventional CT scans with volumetric evaluation of the periablational safety margin was performed using a non-rigid registration software (Ablation-fit^TM^; R.A.W. Srl). At first, anonymized pre- and post-interventional CT scans, acquired during the stereotactic RFA procedure, were imported into the software. Both consisted of a late arterial and portal venous phase contrast-enhanced CT scan (Siemens SOMATOM Sensation Open, sliding gantry with 82-cm diameter, Siemens AG) with 3-mm slice thickness. The CT scans were acquired 35–40 and 70–80 s after injection of 100–150 ml contrast media (Iopromide [Ultravist 370; Schering AG]). After an automatic segmentation of the liver parenchyma, liver metastasis and necrosis zone were semi-automatically segmented and reconstructed. Portal venous phase was utilized as the image of choice for both segmentation of the liver metastasis and necrosis zone. If the semi-automatic segmentation was deemed unsatisfactory, manual modification in the 2D axial visualization was performed in order to achieve an adequate segmentation. Thereafter, the segmented pre- and post-interventional CT scans were automatically registered using a non-rigid registration tool implemented in the software. Using a non-rigid registration tool, liver parenchyma can be deformed section by section, based upon reference to intrahepatic landmarks such as vessels in order to guarantee an exact image fusion result regardless of differences in body position, respiratory motion, or liver deformation due to large necrosis zones. With this exact registration, the software is capable of verifying whether the necrosis zone circumscribes entirely the tumor alone with or without a safety margin. Furthermore, the software rapidly calculates the percentages of residual unablated volumes of both the target tumor and predetermined 3D safety margin in near real-time. The whole image fusion using the current software version of Ablation-fit^TM^ takes an average time of 10 min (5–20 min), depending on the amount of manual correction required. The residual percentages of unablated 3D safety margin were calculated as follows: (volume of the calculated safety margin outside of the necrosis zone divided by the calculated volume of total safety margin surrounding target tumor) × 100.

**Fig. 2:**
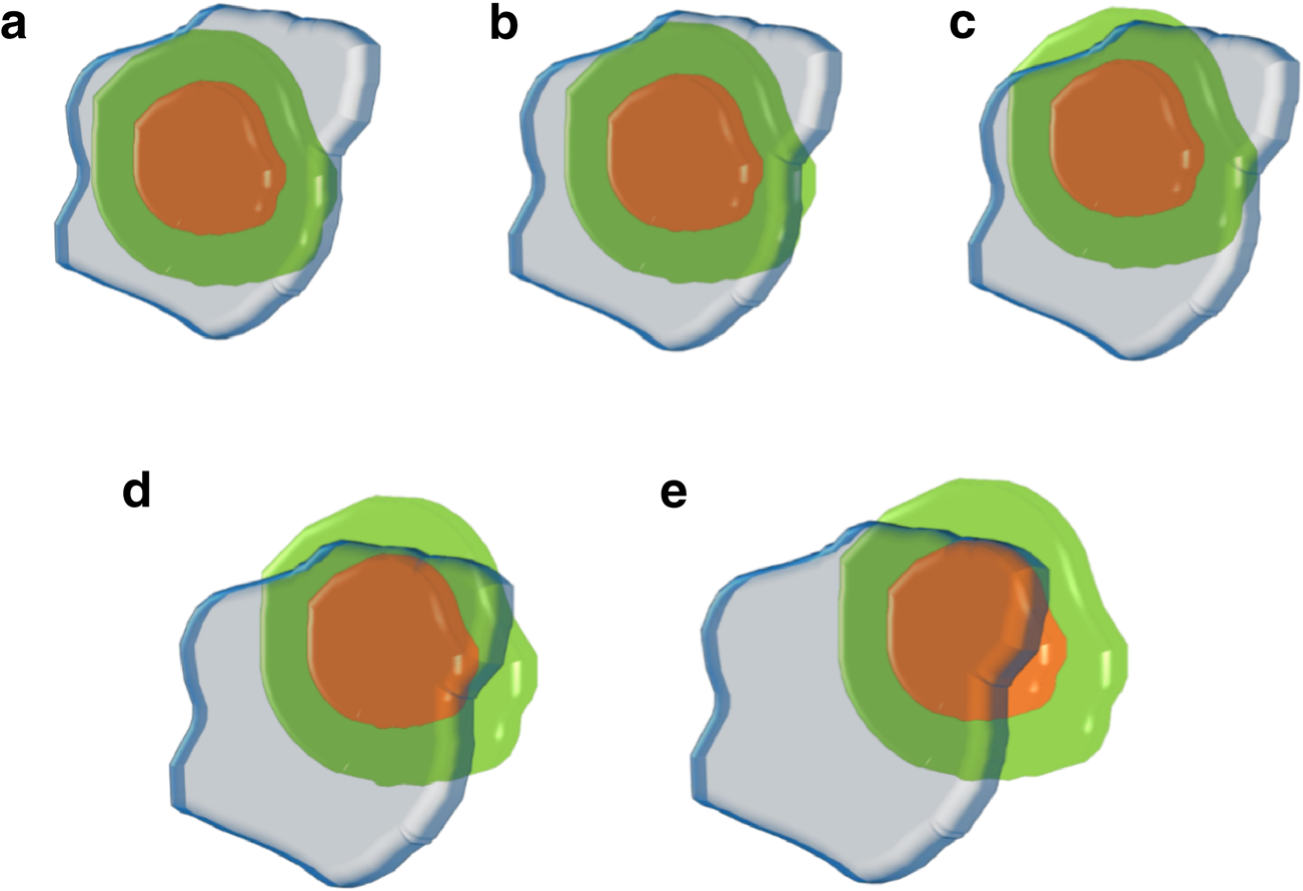
Diagram to illustrate the classification of periablational 3D safety margin (SM) with Ablation-fit^TM^: tumor (orange), predetermined safety margin (green), and necrosis zone (blue). Complete ablation with 100% 3D SM; unablated SM volume = 0% (**a**), complete ablation with 95–100% 3D SM; unablated SM volume < 5% (**b**), complete ablation with 90–95% 3D SM; unablated SM volume = 5–10% (**c**), complete ablation with < 90% 3D SM; unablated SM volume > 10% (**d**), and incomplete ablation with residual tumor (**e**)

**Fig. 3:**
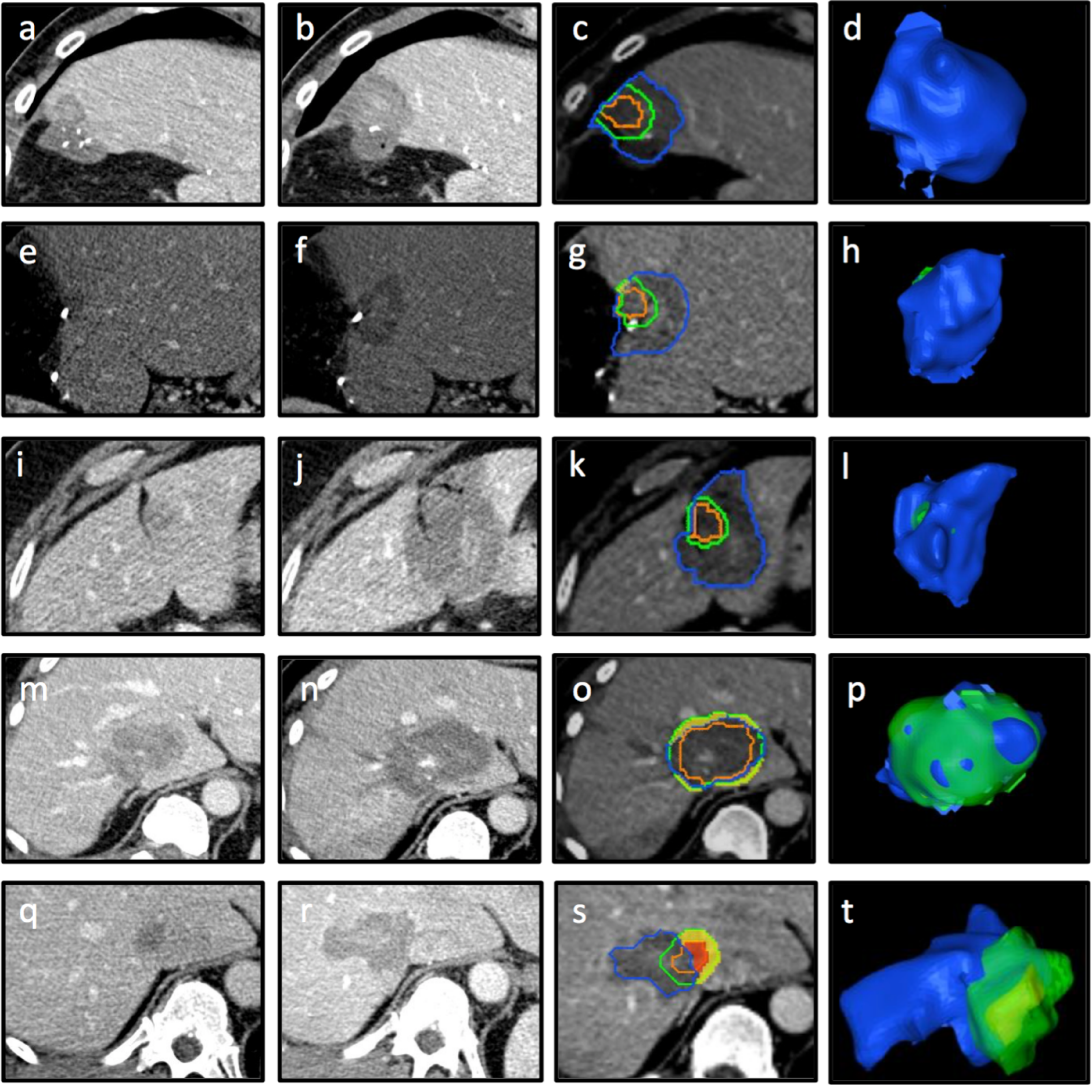
Examples of periablational 3D safety margin (SM) assessment with Ablation-fit^TM^. Complete ablation with 100% 3D SM (**a–d**), 95–100% 3D SM (**e–h**), 90–95% 3D SM (**i**–**l**), < 90% 3D SM (**m–p**), and incomplete ablation with residual tumor (**q–t**). **a**, **e**, **i**, **m** Pre-interventional CT-scan. **b**, **f**, **j**, **n** Post-interventional CT-scan. **c**, **g**, **k**, **o** Non-rigid registration of pre- and post-interventional CT scans with assessment of 3D SM of 5 mm: tumor (orange line), 5-mm safety margin (green line), necrosis zone (blue line), unablated safety margin (yellow area); unablated tumor (red area). **d**, **h**, **l**, **p** 3D reconstruction of necrosis zone (blue), unablated 3D safety margin (green), and unablated residual tumor (yellow)

For this analysis, unablated volume percentages were calculated and separately analyzed. This included 1-, 2-, 3-, 4-, 5-, 6-, 7-, 8-, 9-, and 10-mm periablational 3D safety margins for each of the five classifications: (1) complete ablation with 100% 3D safety margin; (2) complete ablation with 95–100% 3D safety margin; (3) complete ablation with 90–95% 3D safety margin; (4) complete ablation with < 90% 3D safety margin; and (5) incomplete ablation with residual unablated tumor volume. A diagram illustrating the classifications is shown in Fig. 2 and an example of the assessment for each classification with 5 mm as the predetermined 3D safety margin is shown in Fig. 3.

In a second step, for each safety margin value, unablated volume percentages were divided into three categories. Unablated safety margin volume percentages where no LTP was observed were defined as “green,” and percentages with LTP as “red.” Percentages that yielded both LTP and no LTP were labeled as “gray.”

The evaluation of the safety margin using this software was conducted blinded regarding the oncological outcome.

### Statistical analysis

Based on previously published data by Wang et al [8], prior to initiating the study, we performed a sample size power calculation in order to determine how many liver metastases (irrespective of the number of patients) needed to be included to detect a possible difference in LTP between ablations with previously reported sufficient (> 5 mm) and insufficient safety margins (< 5 mm) [7–9, 14]. Assuming a type 1 error of 0.05 and a type 2 error of 0.2 (thus yielding 80% statistical power), we determined that a total of 60 tumors had to be analyzed to detect such a difference.

The distribution (parametric/non-parametric) of all variables was assessed using histograms. Data were expressed as mean ± SD or median and range, as appropriate. Categorical variables were compared with *χ*^2^ test and Fisher’s exact test, respectively, while continuous variables were compared using unpaired, two-sided Student’s *t* test. LTP-free survival probabilities were estimated using Kaplan-Meier survival analysis and compared with the log-rank test. Diagnostic ability was assessed with receiver operating characteristic (ROC) analysis. The ROC analysis results were interpreted as follows: area under the curve (AUC) < 0.70 = low predictive accuracy; AUC in the range of 0.70–0.90 = moderate predictive accuracy; and AUC ≥ 0.90 = high predictive accuracy.

All statistical analyses were performed using SPSS Version 22 (SPSS Inc.). *p* values < 0.05 were considered statistically significant.

## Results

### Patient characteristics

Forty-five patients (14 females/31 males) with mean age of 64.5 years (31–87) and a total of 76 CRLM were included in the current study. Among these individuals, 18 had a solitary liver metastasis, 19 had two tumors, four patients had three metastases, and two patients presented with four metastases. Thirty-one patients had undergone chemotherapy prior to stereotactic RFA, while 15 patients had received prior surgical liver resection. Eighteen patients developed distant tumor recurrence over the course of their disease, with seven of them successfully re-treated with stereotactic RFA. Extrahepatic metastases were observed in 14 patients, with lung metastases being the most prevalent accounting for eleven (78.6%) of the extrahepatic manifestations. In total, 24 patients received adjuvant therapy, of whom 23 underwent adjuvant chemotherapy and one patient was treated with liver resection.

The mean tumor size was 24.2 mm (3–75 mm), whereby 49 (64.5%) tumors were < 30 mm and 27 (35.5%) ≥ 30 mm. The median number of coaxial needles per tumor used for stereotactic RFA was 4 (1–12). Eleven (14.5%) tumors had direct proximity to an extrahepatic organ, 25 (32.9%) tumors were subcapsular in location, and 23 (30.3%) tumors were adjacent to a major intrahepatic vessel. The mean overall follow-up period per tumor was 36.1 ± 18.5 months.

### Local tumor progression

**Fig. 4:**
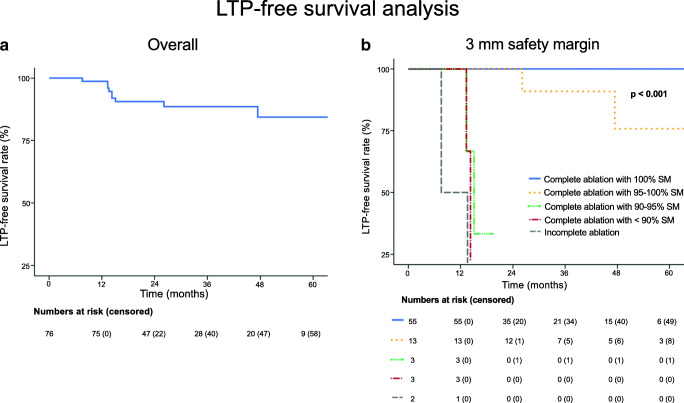
Kaplan-Meier curves of overall LTP-free survival and periablational 3D safety margin assessment with 3 mm

Nine of 76 tumors (11.8%) developed LTP at follow-up with a mean time to LTP of 18.3 ± 11.9 months. A detailed description of those tumors is shown in Table 1. The overall 1-, 2-, and 3-year cumulative LTP-free survival rates were 98.7%, 90.6%, and 88.6%, respectively (Fig. 4a). The evaluation of the periablational 3D safety margin was the only significant predictor of LTP (*p* < 0.001) for all calculated, predetermined 3D safety margins (Table 2). The risk for LTP based on ablation completeness is shown in Table 3. Age, gender, tumor size, tumor location (proximity to major vessel/extrahepatic organ, subcapsular), previous therapies, and adjuvant chemotherapy had no significant influence on LTP rate (Table 4).

**Table 1:** Detailed description of tumors with LTP

#	Age	Gender	Tumor size (mm)	Tumor location	Previous therapy	Time to LTP (months)	3D SM assessment with Ablation-fit^TM^										
							Residual tumor %	Unablated SM %									
								10 mm	9 mm	8 mm	7 mm	6 mm	5 mm	4 mm	3 mm	2 mm	1 mm
**77**	64	Female	32	Subcapsular | major vessel | extrahepatic organ	HR | CTx	7.56	9.1	68.8	65.9	61.8	58.0	53.2	46.1	38.8	30.0	20.7	13.1
58	49	Female	10	Major vessel	HR | CTx	14.29	-	60.4	57.9	55.1	51.9	47.8	42.9	28.6	16.9	0	0
57	49	Female	12	Major vessel	HR | CTx	14.29	-	56.5	53.0	49.5	45.1	39.7	34.9	25.7	14.4	0	0
48	61	Male	20	None	HR | CTx	13.34	-	48.7	44.4	39.2	36.4	31.7	27.1	20.1	14.7	8.0	0
79	62	Male	48	Major vessel	-	15.11	-	42.1	38.6	33.6	29.1	24.5	18.2	13.0	7.6	0	0
13	31	Male	40	None	-	13.60	5.3	38.0	34.5	31.6	28.7	24.6	21.5	19.0	16.2	10.9	7.8
15	69	Male	13	None	-	47.41	-	31.4	29.1	26.8	22.3	16.5	11.6	7.6	4.2	0	0
39	58	Male	36	Subcapsular	HR | CTx	13.34	-	25.1	23.5	21.2	19.2	17.1	15.0	12.3	9.2	5.1	1.3
61	70	Male	30	Major vessel	-	26.12	-	23.3	21.0	15.8	13.4	11.3	6.1	4.4	3.3	0	0

*SM*, safety margin; *HR*, hepatic resection; *CTx*, chemotherapy

**Table 2:** Univariate analysis of different predetermined 3D safety margins as possible risk factors for LTP.

Predetermined SM (mm)	Assessment with Ablation-fit^TM^										*p* value
	100% SM		95–100% SM		90–95% SM		< 90% SM		Residual tumor		
	Total (LTP)	*%*	Total (LTP)	*%*	Total (LTP)	*%*	Total (LTP)	*%*	Total (LTP)	%	
10	8 (0)	0	7 (0)	0	16 (0)	0	43 (7)	16.3	2 (2)	100	*< 0.001*
9	9 (0)	0	11 (0)	0	14 (0)	0	40 (7)	17.5	2 (2)	100	*< 0.001*
8	13 (0)	0	10 (0)	0	20 (0)	0	31 (7)	22.6	2 (2)	100	*< 0.001*
7	14 (0)	0	14 (0)	0	20 (0)	0	26 (7)	26.9	2 (2)	100	*< 0.001*
6	18 (0)	0	19 (0)	0	20 (0)	0	17 (7)	41.2	2 (2)	100	*< 0.001*
5	34 (0)	0	11 (0)	0	18 (1)	55.5	11 (6)	54.5	2 (2)	100	*< 0.001*
4	43 (0)	0	16 (1)	6.3	10 (1)	10	5 (5)	100	2 (2)	100	*< 0.001*
3	55 (0)	0	13 (2)	15.4	3 (2)	66.7	3 (3)	100	2 (2)	100	*< 0.001*
2	70 (5)	7.1	2 (0)	0	2 (2)	100	-	-	2 (2)	100	*< 0.001*
1	73 (6)	8.2	1 (1)	100	-	-	-	-	2 (2)	100	*< 0.001*

Significant values (*p* ≤ 0.05) are italicized

*SM*, safety margin

**Table 3:** Risk for LTP based on ablation completeness

Predetermined SM (mm)	Assessment with Ablation-fit^TM^							
	100% SM		> 95% SM		> 90% SM		All ablations	
	Total (LTP)	%	Total (LTP)	%	Total (LTP)	%	Total (LTP)	%
10	8 (0)	0	15 (0)	0	31 (0)	0	76 (9)	11.8
9	9 (0)	0	20 (0)	0	34 (0)	0	76 (9)	11.8
8	13 (0)	0	23 (0)	0	43 (0)	0	76 (9)	11.8
7	14 (0)	0	28 (0)	0	48 (0)	0	76 (9)	11.8
6	18 (0)	0	37 (0)	0	57 (0)	0	76 (9)	11.8
5	34 (0)	0	45 (0)	0	63 (1)	1.6	76 (9)	11.8
4	43 (0)	0	59 (1)	1.7	69 (2)	2.9	76 (9)	11.8
3	55 (0)	0	68 (2)	2.9	71 (4)	5.6	76 (9)	11.8
2	70 (5)	7.1	72 (5)	6.9	74 (7)	9.4	76 (9)	11.8
1	73 (6)	8.2	74 (7)	9.4	-	-	76 (9)	11.8

*SM*, safety margin

**Table 4:** Univariate analysis of possible risk factors for LTP in 76 tumors

Variable	No LTP	LTP	*p* value
Gender			0.974
Male	46	6	
Female	21	3	
Age			0.238
> 60 years	49	5	
< 60 years	18	4	
Tumor size			0.132
< 30 mm	45	4	
≥ 30 mm	22	5	
Subcapsular location			0.565
Yes	23	2	
No	44	7	
Proximity to extrahepatic organ			0.284
Yes	9	2	
No	58	7	
Proximity to major vessel			0.094
Yes	18	5	
No	49	4	
CTx prior to stereotactic RFA			0.232
Yes	50	5	
No	17	4	
HR prior to stereotactic RFA			0.109
Yes	19	5	
No	48	4	
Adjuvant CTx			0.133
Yes	34	7	
No	33	2	

*LTP*, local tumor progression; *CTx*, chemotherapy; *HR*, hepatic resection

The smallest safety margin displaying no LTP in ablations with a 100% circumscribed 3D safety margin was 3 mm with 0/55 (0%) tumors. Correspondingly, 3-year LTP-free survival rates for ablations with 100% safety margin, 95–100% safety margin, 90–95% safety margin, < 90% safety margin, and incomplete ablation using 3 mm as the safety margin were 100%, 91%, 0%, 0%, and 0%, respectively (*p* < 0.001; Fig. 4b). A safety margin of 6 mm was the smallest distance with no LTP in ablations with at least 90% safety margin (Tables 2, 3). Regardless of the distance, ablations with < 90% circumscribed periablational safety margin showed high LTP rates, ranging from 16.3% (10 mm) to 100% (3 mm) as shown in Table 2.

**Fig. 5:**
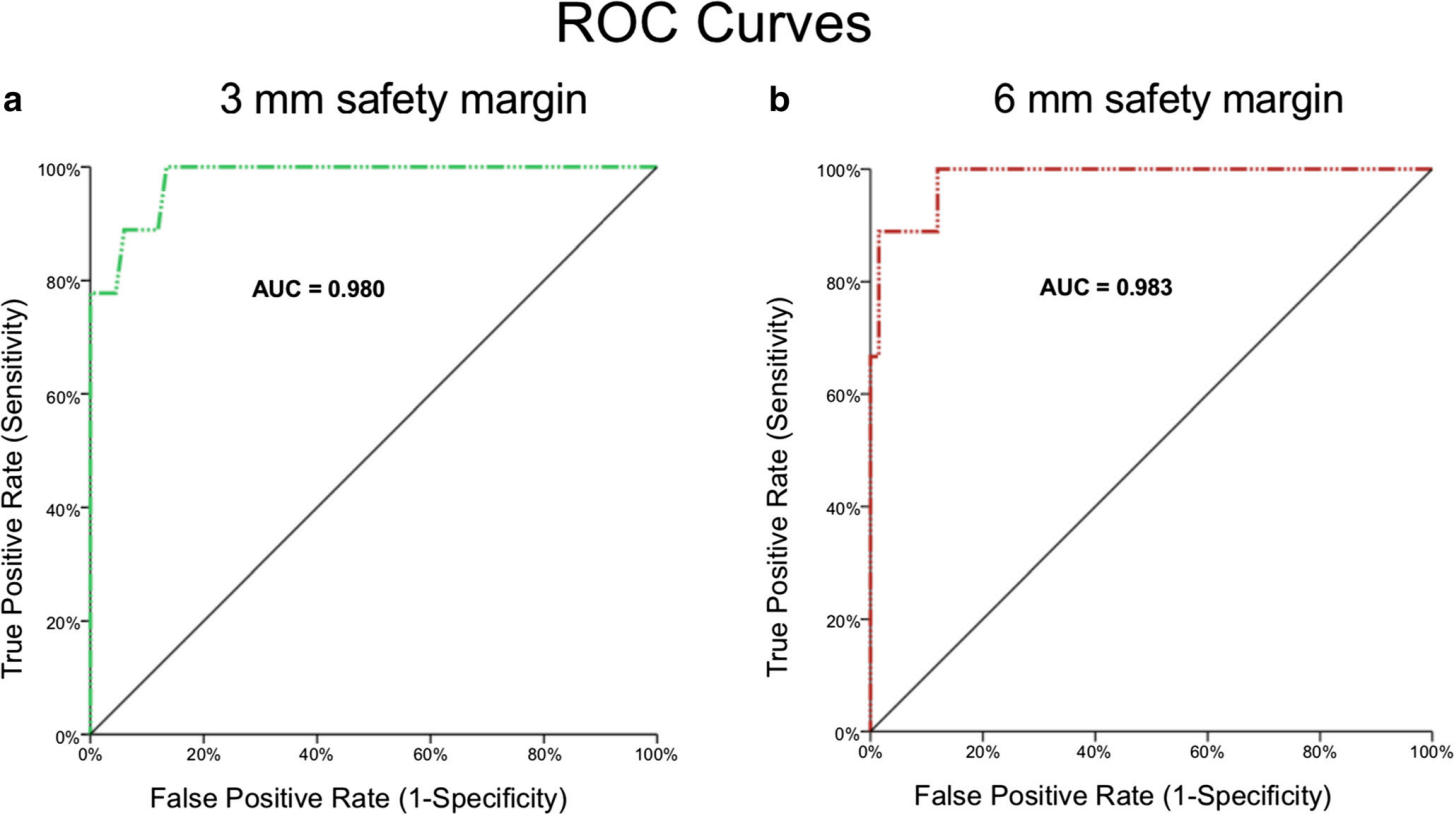
ROC curves for 3 and 6 mm as periablational 3D safety margins

ROC analysis revealed a high accuracy (AUC > 0.9) in predicting LTP for all calculated safety margins, except for 2 and 1 mm. ROC curves for 3 and 6 mm as the predetermined safety margins are shown in Fig. 5. Thresholds of unablated safety margin percentages with 100% sensitivity in predicting successful ablations for 3 and 6 mm were 3.2% and 11.2%, respectively. The related specificities were 86.6% (3 mm) and 88.1% (6 mm).

**Fig. 6:**
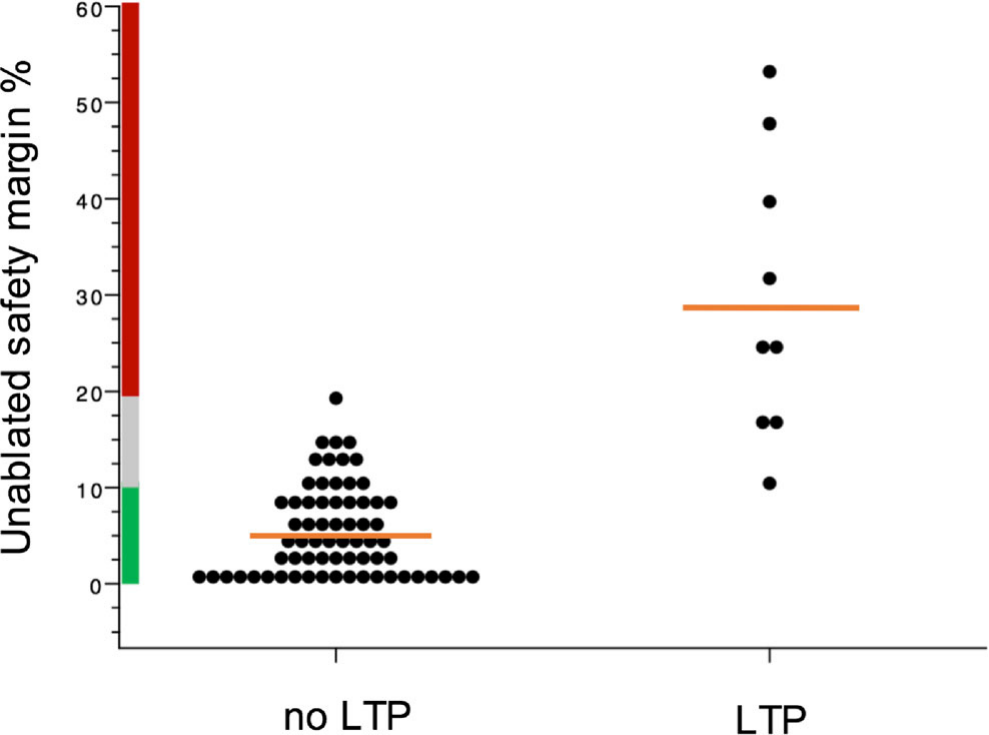
Dot plot with percentages of unablated 3D safety margin volumes for 6 mm in target tumors with and without LTP (mean = orange line). On the y-axis division of unablated volume percentages in categories: “green” (no LTP), “gray” (LTP possible), and “red” (LTP). Each dot represents one target tumor

The mean percentages of unablated 3D safety margin volumes were significantly higher in tumors with LTP (*p* < 0.001) for all safety margins. Using a 6-mm safety margin, there is an 11.2–19.3% gray zone threshold (Fig. 6).

## Discussion

With this work, we confirm prior reports demonstrating the importance of achieving a periablational margin for ablation, as we too established the periablational safety margin as the only significant predictor of LTP in patients with CRLM treated with stereotactic RFA. Apart from the periablational safety margin, no other conventional risk factor such as age, gender, tumor size, tumor location, or previous therapies significantly influenced LTP. Importantly, this observation held true for all calculated 3D safety margins, namely, 1–10 mm, respectively. Yet, in our study the smallest safety margin displaying no LTP for ablations with 100% circumscribed 3D safety margin was 3 mm (0/55 [0%] target tumors). This indicates that an ablation with a 100% circumscribed 3D safety margin of 3 mm can be considered successful at the time of the intervention. Given that larger volumes of ablation are more difficult to achieve, this may represent an improvement when considering that previous studies [7–9] recommend a periablational safety margin of at least 5 mm, with better results only seen with a 10-mm periablational safety margin for CRLM. One possible explanation for this discrepancy might be the exact image fusion and volumetric safety margin assessment achieved with the non-rigid registration software used for this study. Regardless, the small but precise margins in our study may actually represent larger volumes given known tissue shrinkage during ablation [20].

Our data also suggests that ablations with at least 90% circumscribed 3D safety margin of 6 mm can also be considered sufficient. Thus, it may not be strictly necessary to retreat unablated volumes in cases where circumscribed 100% safety margin cannot be guaranteed due to unavoidable influencing factors (e.g., subcapsular location or major vessel vicinity). This information may increase confidence in not immediately retreating nearly complete margins and lower the additional risk for complications due to potentially unnecessary secondary procedures. On the other hand, ablations with less than 90% circumscribed 3D safety margin have to be considered unsuccessful due to high LTP rates, regardless of the distance.

Several different approaches for safety margin assessment in primary and secondary liver malignancies have been proposed by the scientific community [8, 13, 21–24]. Furthermore, Kaye et al [14] recently demonstrated that a volumetric 3D assessment of the periablational safety margin had a higher LTP discrimination power compared to a manual 2D approach. Nevertheless, most institutions neither use a 3D nor a 2D assessment of the periablational safety margin, but rather rely on the conventional approach: side-by-side juxtaposition of pre- and post-interventional CT scans with evaluation of treatment success by visual inspection. This conventional approach is very challenging even for experienced radiologists and may introduce several potential sources of error including differences in body position, respiratory motion, or liver deformation due to large necrosis volumes. Using a non-rigid registration tool, liver parenchyma can be deformed section by section, referring to intrahepatic landmarks such as vessels. Thus, limitations of the conventional approach can be overcome by better more accurate fusion matching.

The time required for such an evaluation remains a crucial factor before introducing it into clinical routine. The non-rigid registration software utilized in the present study is user-friendly and focuses only on essential calculations. From our experience, only 10 min is needed for the entire calculation and display. The assessment can also easily be split, conducting the segmentation of the target tumor during/before ablation and the segmentation of the necrosis zone with subsequent image fusion once the control CT has been obtained. In contrast to registration tools that are already implemented in diagnostic viewing programs, this non-rigid registration software including its quantitative assessment of the ablation margin is fully automatic. This can save time and potentially assures an objective, non-biased image fusion. The result of the image fusion can then be verified by the performing radiologist using a “registration blending” function, switching from pre- to post-interventional CT scan. Furthermore, the visual presentation of unablated safety margin volumes allows an extension of the ablation in the same session, if needed.

Overall, our data revealed a relatively low LTP rate (11.8%), which is situated at the bottom end of the range compared to previous studies [10]. In comparison to another study assessing the periablational safety margin in patients with CRLM treated with RFA [8], considerably lower overall LTP-free survival rates were achieved in the current study using stereotactic RFA (1-, 2-, 3-year cumulative LTP-free survival rates: 98.7% vs. 59%, 90.6% vs. 46%, and 88.6% vs. 38%, respectively). These observations could be explained by the stereotactic approach, which has already proven successful for treating ablations in large or difficult to reach liver malignancies [11, 25, 26]. Whereas tumor size is frequently considered a critical determinant for ablation success [8, 27], it had no impact on LTP in our cohort. This is a remarkable finding given that tumors treated in the current study exhibited diameters of up to 75 mm, and prior reports noted higher levels of recurrence for tumors exceeding 3 and certainly 5 cm [10].

Major limitations of our study lie in its retrospective design and the single-center bias. Moreover, the number of patients included with unsuccessful ablations was relatively small. Another limitation is certainly the current restriction of the presented software that limits image fusion to pairs of CT images. Therefore, an expansion of the software capabilities to include fusion with other modalities such as MRI would be desirable and may play an important role for future studies. Furthermore, we acknowledge that additional validation of the software for a wider spectrum of ablation procedures including a wider range of ablation devices and techniques is needed. The comparison of our results with similar studies is hampered by the scarce use of stereotactic approaches in the ablation of liver malignancies elsewhere than in our own institution. Nevertheless, our findings are encouraging. Thus, we hope that this study aids in generating further interest in the technique.

In conclusion, our study has three important implications: (1) Volumetric assessment of the periablational safety margin can be effectively used as an intraprocedural tool to rapidly and accurately evaluate local treatment success in patients with CRLM referred to stereotactic RFA. (2) Ablations with 100% 3D periablational safety margin of 3 mm and ablations with at least 90% 3D safety margin of 6 mm can be considered indications of treatment success. (3) Image fusion of pre- and post-interventional CT scans with the non-rigid registration software presented in this study is feasible and might represent a useful tool in daily clinical practice.

## Abbreviations

CRLM: Colorectal liver metastases
LTP: Local tumor progression
RFA: Radiofrequency ablation
SM: Safety margin
