# Correction to: Volumetric assessment of the periablational safety margin after thermal ablation of colorectal liver metastases

**DOI:** 10.1007/s00330-021-08107-1

**Published:** 2021-06-17

**Authors:** Gregor Laimer, Nikolai Jaschke, Peter Schullian, Daniel Putzer, Gernot Eberle, Marco Solbiati, Luigi Solbiati, S. Nahum Goldberg, Reto Bale

**Affiliations:** 1 Interventional Oncology-Microinvasive Therapy (SIP), Department of Radiology, Medical University Innsbruck, Anichstr. 35, 6020 Innsbruck, Austria; 2 Department of Internal Medicine I, Gastroenterology, Hepatology, Endocrinology and Metabolism, Medical University Innsbruck, Anichstr. 35, 6020 Innsbruck, Austria; 3 Division of Endocrinology and Metabolic Bone Diseases, Department of Medicine III, Technical University of Dresden, Fetscherstr. 74, 01037 Dresden, Germany; 4 R&D Unit, R.A.W. Srl, Milan, Italy; 5 Department of Biomedical Sciences, Humanitas University, Via Rita Levi Montalcini 4, 20072 Pieve Emanuele, Milan, Italy; 6 IRCCS Humanitas Research Hospital, via Manzoni 56, 20089 Rozzano, Milan, Italy; 7 Department of Radiology, Hadassah Hebrew University Medical Centre, Jerusalem, Israel; 8 Department of Radiology, Beth Israel Deaconess Medical Center, Boston, MA USA

**Correction to: European Radiology.**

10.1007/s00330-020-07579-x

The original version of this article, published on 14 January 2021, unfortunately contained a mistake. The following correction has therefore been made in the original: The affiliations 5 and 6 were presented incorrectly. The corrected affiliations are given below. The original article has been corrected.

